# Satisfaction and Quality of Life of Healthy and Unilateral Diseased *BRCA1/2* Pathogenic Variant Carriers after Risk-Reducing Mastectomy and Reconstruction Using the BREAST-Q Questionnaire

**DOI:** 10.3390/genes13081357

**Published:** 2022-07-28

**Authors:** Natalie Herold, Martin Hellmich, Frank Lichtenheldt, Beyhan Ataseven, Vanessa Hillebrand, Barbara Wappenschmidt, Rita Katharina Schmutzler, Kerstin Rhiem

**Affiliations:** 1 Center for Familial Breast and Ovarian Cancer, Center for Integrated Oncology (CIO), Faculty of Medicine, University Hospital of Cologne, 50937 Cologne, Germany; natalie.herold@uk-koeln.de (N.H.); frank.lichtenheldt@gmx.net (F.L.); vhilleb2@smail.uni-koeln.de (V.H.); barbara.wappenschmidt@uk-koeln.de (B.W.); rita.schmutzler@uk-koeln.de (R.K.S.); 2 Institute of Medical Statistics and Computational Biology, Faculty of Medicine and University Hospital Cologne, University of Cologne, 50924 Cologne, Germany; martin.hellmich@uni-koeln.de; 3 Department of Obstetrics and Gynecology, University Hospital, LMU Munich, 81377 Munich, Germany; b.ataseven@kem-med.com; 4 Evangelische Kliniken Essen Mitte, 45136 Essen, Germany

**Keywords:** *BRCA1*, *BRCA2*, breast cancer, risk-reducing mastectomy, BREAST-Q

## Abstract

Risk-reducing mastectomy (RRM) is the most efficient form of breast cancer (BC) risk reduction in *BRCA1/2* pathogenic variant (pV) carriers. However, this intervention in physical integrity is associated with significant morbidity. We assessed long-term perception of satisfaction and health-related quality of life (QoL) after bilateral RRM and reconstruction using the validated BREAST-Q. We searched the prospective database of the Center for Hereditary Breast and Ovarian Cancer Cologne for previvors and survivors who underwent bilateral RRM from 1994 to 2015 and evaluated the results of their BREAST-Q scores. The study enrolled 43 previvors and 90 survivors after a mean follow-up of 46.3 ± 45.3 months after RRM. Satisfaction and QoL were independent of the technique of RRM or type of reconstruction but depended on the time of RRM. Compared to survivors, previvors had significantly higher mean satisfaction scores in their psychosocial, sexual, and physical well-being (chest) in both modules. Among previvors and survivors, higher psychological well-being correlated with a higher satisfaction with information and higher satisfaction with outcome. As psychological well-being correlated with satisfaction with information and outcome, we developed decision aids to improve shared decision making and long-term satisfaction with the decision and the postoperative outcome.

## 1. Introduction

Women carrying a pathogenic variant (pV) in *BRCA1/2* face elevated lifetime risks for breast cancer (BC), contralateral BC, and ovarian cancer (OC). Large cohort studies estimate a cumulative BC risk to age 80 years of 72% for *BRCA1* and 69% for *BRCA2* pV carriers, for contralateral BC of 20–25% for *BRCA1* and 7–13% for *BRCA2* pV carriers 10 years after first BC [1], and 40% for *BRCA1* and 26% for *BRCA2* pV carriers 20 years after first BC [2]. The cumulative OC risk to age 80 was 44% for *BRCA1* and 17% for *BRCA2* pV carriers [3].

Risk-adapted preventive measures range from intensified BC surveillance to risk-reducing mastectomy (RRM). Bilateral RRM significantly reduces BC risk in previvors [4,5]. Contralateral RRM (CRRM) significantly decreases contralateral BC incidence in both *BRCA1/2* pV carriers [6] and reduces overall and BC-specific mortality rates for *BRCA1* pV carriers [7].

Methods of mastectomy span simple, radical, nipple-sparing (NSM), subcutaneous (SCM), skin-sparing (SSM), and total-skin-sparing mastectomy [8,9]. The reconstruction process requires making numerous clinical decisions ranging from the timing (immediate/delayed-immediate/never) to implant-based (IBR) and autologous-based breast reconstruction (ABR).

However, these surgeries are associated with significant morbidities, especially considering the benefit of MRI breast screening for previvors with *BRCA1/2* pV. Prospective cohort studies demonstrated that MRI breast screening allows for early BC detection [10,11,12] and provides first indications for a survival benefit for *BRCA1* pV carriers [13].

The evolution of personalized genomic oncology leads to an increase in (predictive) genetic testing and to an awareness of the complexity of the preventive options. Manifold guidelines recommend the participation of patients in shared decision making (http://www.leitlinienprogramm-onkologie.de/leitlinien/mammakarzinom/ (accessed on 20 June 2022; Deutsche Krebsgesellschaft DKG).

Decision aids for pV carriers must include patient-reported satisfaction and quality of life (QoL) after surgery, especially as surgeons and patients can assess perceived aesthetic outcomes and satisfaction with reconstruction differently [14].

However, study data for previvors and survivors with *BRCA1/2* pV exclusively using the BREAST-Q [15,16] are rare, whereas other groups investigated previvors only, previvors and survivors with pV in *BRCA1/2*, and further BC disposition genes or high-risk families without pV [17,18,19,20].

## 2. Materials and Methods

This project was approved by the Ethics Committee of the University Hospital of Cologne (07-048).

The prospective database of the Center for Familial Breast and Ovarian Cancer Cologne, which is part of the database of the German Consortium for Hereditary Breast and Ovarian Cancer (GC-HBOC), was retrospectively reviewed to identify previvors and survivors who underwent BRRM. We identified 250 women, whose data were collected and documented and who had agreed to be contacted for accompanying scientific projects. All patients fulfilled the inclusion criteria for genetic testing defined and validated by the GC-HBOC [21]. All participants carried a pV in *BRCA1* or *BRCA2* classified according to the IARC system [22], which was based on the guidelines of the ENIGMA Consortium [23], of the American College of Medical Genetics and Genomics (ACMG), and of the Association for Clinical Genomic Science (ACGS). They were incorporated into the guidelines of GC-HBOC. Surgical techniques were obtained from patients’ files. Surgeries were performed at the University Hospital of Cologne or elsewhere between 1994 and 2015.

A total of 250 study documents were dispatched between September 2015 and April 2016. They contained a German version of the BREAST-Q mastectomy and reconstruction questionnaire with 116 questionnaire items. Each scale produces an independent score from 0 to 100, with a higher score meaning higher satisfaction. The BREAST-Q mastectomy module was considered not valid if immediate reconstruction had been performed.

**Figure 1:**
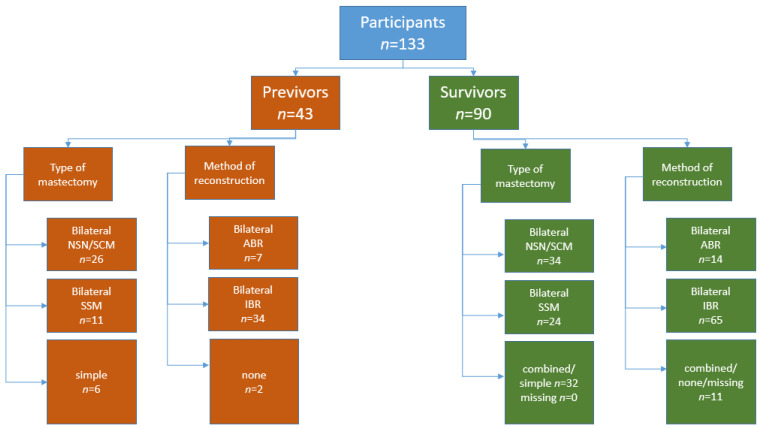
Cohort diagram. Study participants and method of mastectomy and reconstruction. Missing indicates number of missing information on RRM. Legend: ABR: autologous-based breast reconstruction, IBR: implant-based breast reconstruction, *n* = number, NSM/SCM nipple sparing/ subcutaneous mastectomy, SSM: skin-sparing mastectomy.

The following demographic data were noted in all participants: year of birth, genetic status, age at RRM, type of mastectomy and reconstruction (ABR, IBR or combined), nipple reconstruction and date of the most recent reconstructive surgery (definite reconstruction or surgical correction), performance, and date of risk-reducing salpingo-oophorectomy (RRSO). Patient characteristics are summarized in Table 1 and Figure 1.

Additionally, we documented the following items in survivors: age of diagnosis, laterality, histology, cancer stage, method of surgery of the affected breast, receipt, and date of (neo-) adjuvant chemotherapy, endocrine therapy, targeted therapy, and radiation. Follow-up time was defined as time after last reconstructive surgery.

We documented postoperative scales of the BREAST-Q mastectomy and reconstruction modules on health-related quality of life (HR-QoL, including physical, psychosocial, and sexual well-being) and patient satisfaction with breasts, outcome, and care.

Quantitative variables were summarized by mean ± standard deviation, and qualitative variables by absolute and relative (%) frequencies. Data distributions of satisfaction and QoL scores (range zero to 100) were tested for deviations from normality (visual inspection, Shapiro–Wilk test). The Mann–Whitney *U*-test was used to compare distributions of scores between healthy carriers and patients, as well as between different methods of mastectomy and reconstruction. Linear regression was used to describe the relationship (and correlation) between scores. All calculations were performed using SPSS Statistics software (IBM Corp., Armonk, NY, USA). All *p*-values are two-tailored. Values of *p* ≤ 0.05 were considered statistically significant (“experiment-wise”).

## 3. Results

### 3.1. Overall Cohort

A total of 133 of 250 contacted *BRCA1/2* pV carriers resent the BREAST-Q questionnaires (response rate 53.2%). In total, 122 participants (91.7%) completed all four modules of the questionnaire. Among them were 85 *BRCA1* (63.9%) and 48 *BRCA2* pV carriers (36.1%), 43 previvors and 90 survivors. Previvors were significantly younger at RRM than survivors: 36.4 ± 7.9 years (23–51 years) vs. 41.9 ± 8.2 years (21–60 years) (*p* = 0.000).

Mean age at last surgery (definite or corrective surgery) was 40.1 ± 8.5 years (21–61 years). Mean follow-up time after last surgery was 46.3 ± 45.3 months (range 4.2–253.0 months, 75%CI 41.8–50.9 years). Mean follow-up for previvors was shorter than for survivors (43.3 ± 33.3 vs. 47.8 ± 50.2 months). This difference was not significant (*p* = 0.595). Surgical complications were obtained from patients’ files and occurred in 17% of the participants (capsular fibrosis, flap necrosis). Planned re-surgeries (e.g., aesthetic correction) were not rated as complications.

A total of 48 of 113 participants (36.1%) decided on a RRSO. Mean age was 46.6 ± 6.4 years. Previvors were younger than survivors (41.5 ± 6.5 years (*n* = 12) vs. 44.0 ± 6.7 years (*n* = 36)). The difference was not significant (*p* = 0.264).

One BC occurred in a *BRCA1* pV carrier 62 months after BRRM at the age of 43. Her BREAST-Q scores were obtained 22 months after surgery of the unilateral BC diagnosis.

### 3.2. Previvors

Among the 43 previvors were 25 *BRCA1* and 18 *BRCA2* pV carriers. Mean age at BRRM was 36.4 ± 7.9 years (range 23–51 years). In total, 26 previvors decided on bilateral NS/SCM, 11 on an SSM, and 6 on a simple mastectomy. Thirty-four previvors decided on IBR, seven on ABR, and two refrained from reconstruction.

### 3.3. Survivors

Among the 90 survivors were 60 *BRCA1* and 30 *BRCA2* pV carriers. Mean age at BC diagnosis was 39.6 ± 8 years (range 20–58 years). Mean age at CRRM was 41.9 ± 8.2 years (range 21–60 years) (Table 1).

#### 3.3.1. Tumor Type and Therapy

Initial tumor/nodal stages at BC diagnosis were T1 (*n* = 53), T2 (*n* = 20), T3 (*n* = 2), N0 (*n* = 63), N1 (*n* = 3), N2 (*n* = 3), and N3 (*n* = 1). Histopathological subtypes were triple-negative breast cancer (TNBC) (*n* = 49, 42 in *BRCA1* pV carriers), endocrine sensitive (*n* = 41), and Her2 positive (*n* = 9). A total of 32 of 41 endocrine-sensitive tumors were treated with endocrine therapy. In total, 35 survivors received neoadjuvant chemotherapy, 46 received adjuvant chemotherapy, and nine had no chemotherapy.

#### 3.3.2. Radiation

A total of 36 survivors received adjuvant radiation, 29 after BCT and 7 after mastectomy (Table 2).

#### 3.3.3. Mastectomy and Reconstruction

A total of 34 survivors underwent a bilateral NSM/SCM, and 24 a bilateral SSM; 32 survivors had a combination of both methods and a simple mastectomy.

In total, 65 survivors underwent bilateral IBR, 14 bilateral ABR, and 11 had a combined reconstruction or refrained from reconstruction, or the information was unable to be obtained from the patients’ files; 38 survivors had an initial breast conserving therapy (BCT). Secondary mastectomy was performed in two cases because of residual tumor tissue after BCT, in three cases because of a recurrent disease, and in the other 33 cases for no oncological indication. Among the 90 survivors, 28 underwent initial therapeutic mastectomy with immediate CRRM, whereas 16 underwent initial therapeutic mastectomy with delayed CRRM (Figure 1).

#### 3.3.4. Ovarian Cancer

Two survivors were diagnosed with an incidental OC during RRSO, and two survivors had a history of OC before CRRM.

### 3.4. Overall Results: BREAST-Q Mastectomy and Reconstruction Module

#### 3.4.1. Satisfaction Scores within the Whole Cohort

The follow-up time of the 133 participants amounted to 46.3 ± 45.3 months (range 4.2–253.0 months). Neither the chosen type of mastectomy nor the type of reconstruction had a significant impact on the satisfaction scores.

Both previvors and survivors with a higher score of psychological well-being had a higher satisfaction with information in the reconstruction module. Furthermore, probands with a higher satisfaction with information showed a higher satisfaction with outcome (*p* < 0.001).

#### 3.4.2. Satisfaction Scores According to the Indication for Surgery: Comparison between Previvors and Survivors

Results of the mastectomy module were considered not valid if immediate reconstruction was performed. Results of the reconstruction module after combined reconstruction methods were not evaluated.

Previvors had significantly higher scores compared to survivors in the following modules of mastectomy and in the following modules of reconstruction: psychosocial well-being (*p* = 0.015), physical well-being (chest) (*p* < 0.000), and sexual well-being (*p* = 0.002). They also had a significantly higher satisfaction with information in the reconstruction module: 76.9 ± 16.1 vs. 68.6 ± 17.4 (*p* = 0.014).

#### 3.4.3. Comparison of Scores among Previvors According to the Type of Mastectomy and Type of Reconstruction

In the mastectomy module, previvors reported significantly higher satisfaction with surgeon after autologous (*n* = 6) than after heterologous reconstruction (*n* = 33): 96.05 ± 6.5 vs. 87.6 ± 15.6; *p* = 0.014, and significantly higher satisfaction with medical staff after SSM (*n* = 11) than after NSM/SCM (*n* = 23): 94.5 ± 9.5 vs. 84.9 ± 19.7; *p* = 0.006.

In the reconstruction module, previvors reported significantly higher satisfaction with the surgeon after SSM (*n* = 11) than after NSM/SCM (*n* = 23): 98.3 ± 5.7 vs. 89.4 ± 13.9; *p* = 0.004.

#### 3.4.4. Comparison among Survivors According to the Type of Mastectomy and Type of Reconstruction

Survivors reported significantly higher scores of physical well-being (chest) after NSM/SCM than after SSM (*p* = 0.016) in the mastectomy module. We detected no significant differences in satisfaction and QoL according to the type of reconstruction within our observed follow-up time of 46.3 ± 45.3 months.

### 3.5. Influence of Tumor Therapy

The 81 survivors who received chemotherapy had significantly lower scores in psychological well-being, satisfaction with the surgeon, and satisfaction with the medical staff than the nine survivors without chemotherapy in the mastectomy module (*p* = 0.013, *p* < 0.001, *p* < 0.001, respectively) and the reconstruction module (*p* = 0.003, *p* = 0.018, *p* < 0.001, respectively).

The 36 survivors after radiation had a significantly lower satisfaction with the surgeon in the mastectomy (*p* = 0.037) and reconstruction module (*p* = 0.049) compared to the 54 survivors without radiation. We showed no significant influence of endocrine therapy on any score of the mastectomy or reconstruction module of the BREAST-Q.

Compared to survivors without RRSO (*n* = 54), survivors after RRSO (*n* = 36) had a significantly lower satisfaction with the surgeon in the reconstruction module, but not in the mastectomy module.

Survivors with a more recent reconstruction had a significantly higher satisfaction with the reconstructed nipple (*p* = 0.02; *n* = 19). We showed no significant correlation for any other item of the reconstruction module among previvors and survivors over time (Table 3).

**Figure 2:**
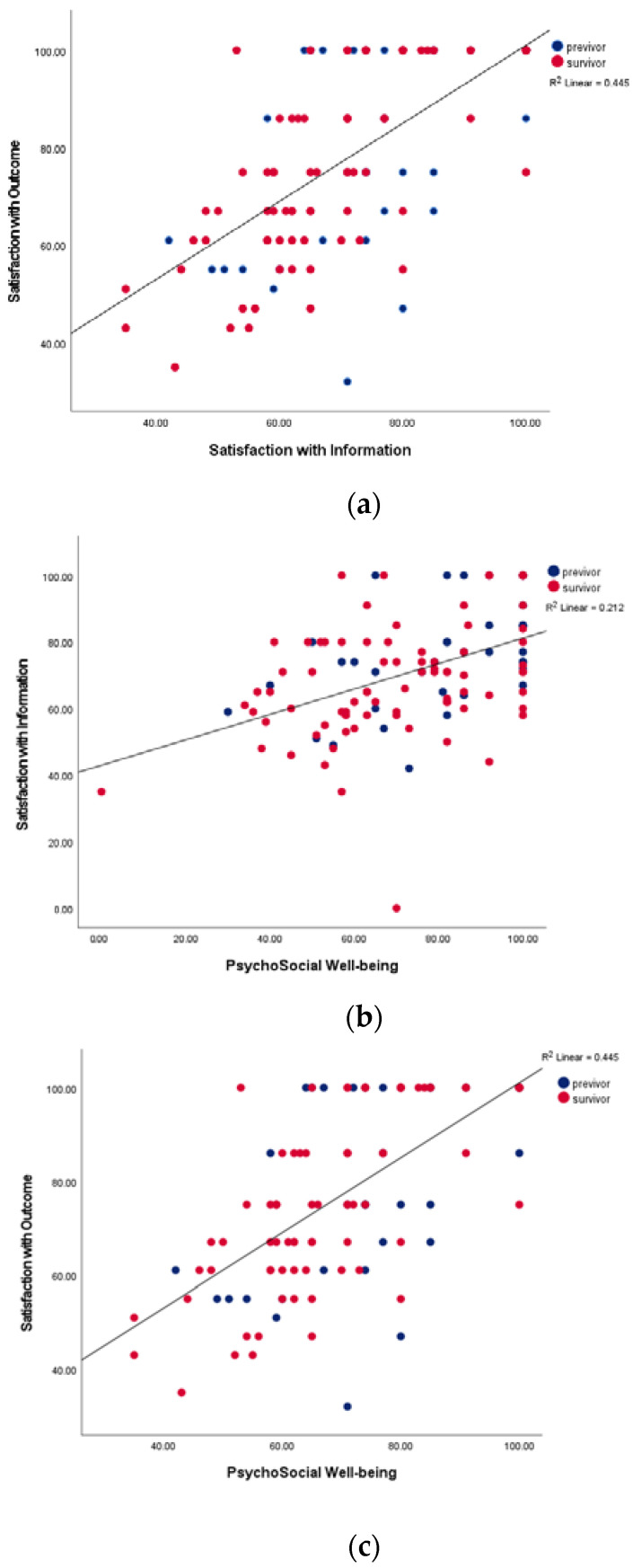
Highly significant correlation of psychological well-being, satisfaction with information, and satisfaction with outcome among all participants: previvors (blue) and survivors (red). (**a**) Satisfaction with outcome and satisfaction with information *p* = 0.000. (**b**) Psychosocial well-being and satisfaction with information, *p* = 0.000. (**c**) Satisfaction with outcome and psychosocial well-being, *p* = 0.000.

Our data suggest that both previvors and survivors with a higher score of psychological well-being had a higher satisfaction with information in the reconstruction module. Furthermore, there was an association between satisfaction with information and satisfaction with outcome (Figure 2a–c).

## 4. Discussion

Carriers of pV in *BRCA1/2* increasingly decide on BRRM and CRRM. The increased demand for genetic testing for cancer risk genes with the decreasing costs for sequencing of multiple genes will lead to a rising number of identified carriers asking for RRM [24].

The rate of previvors opting out for RRM ranged from 34% in Wales between 1995 and 2015 [25], and 44% in Germany between 2009 and 2011 [26]. The rate of 24% more than doubled to 51.8% following the announcement by Angelina Jolie in 2013, with a sustained longer-term effect in the UK [27].

Counselling requires valid data on statistical benefit of RRM, age-related cancer incidences, and patient-reported outcomes on RR surgery [28]. In particular, the Strasser score, a surgeon-reported cosmetic outcome, only poorly reflects the mediocre or poorly perceived cosmetic outcomes in the patient reported BREAST-Q [14].

The statistical benefit of RRM for previvors is adequately proven [6,7] and leads to a risk reduction of 94% [5]. We observed one BC case after RRM within the follow-up time of 1924.6 patient months. The age-adjusted incidences published by Kuchenbaecker et al. would have predicted 2.76 cases during this follow-up time [3], leading to a risk reduction of 62%. Our data suggest that both previvors and survivors with a higher score of psychological well-being had a higher satisfaction with information in the reconstruction module. Furthermore, there is an association between satisfaction with information and satisfaction with outcome (*p* < 0.001).

Among the whole cohort, we detected no significant influence of the method of mastectomy or the method of reconstruction on BREAST-Q scores within the follow-up time of 46.3 ± 45.3 months (range 4.2–253.0 months). In contrast, meta-analyses reported higher satisfaction with overall outcome and breasts after ABR versus IBR [29].

For *BRCA1/2* pV carriers, the indication for surgery (with or without BC diagnosis) appears to be a main influence on satisfaction and QoL.

Previvors had significantly higher scores compared to survivors in the following modules of mastectomy as well as in the following modules of reconstruction: psychosocial well-being (*p* = 0.015), physical well-being (chest) (*p* < 0.001), and sexual well-being (*p* = 0.001). Data were in accordance with 2-year mean patient-reported outcome of the Mastectomy Reconstruction Outcomes Consortium Study. Additionally, previvors stated a significantly higher satisfaction with information after reconstruction 76.9 ± 16.1 vs. 68.6 ± 17.4 (*p* = 0.012).

To improve counselling, our working group developed decision aids and presented advantages and disadvantages of intensive surveillance and methods of RRM and reconstruction [30] for previvors and survivors. Many factors influence the decision-making process with regard to the use of preventive measures, such as individual cancer risk, cancer worry, risk perception, completion of family planning, a first degree affected relative, and having a young child [31].

In our interviews for the development of decision aids, previvors requested more information on RRM and RRSO and psychological aspects [30]. RRM has been associated with lowered general anxiety and reduced concern about breast cancer as early as in 2000 [32]. Van Egdom et al. compared BREAST-Q scores of previvors under intensive surveillance and after RRM. After surgery, previvors had significantly lower scores in physical well-being and higher scores in psychological well-being [33].

Previvors were significantly younger at RRM and younger at study participation. They were tested for the mutation already known in the family with a longer reflection period on testing and preventive options. This may explain the higher satisfaction with information compared to the survivors.

The survivors either underwent genetic testing because of their BC diagnosis or were previvors diagnosed with BC during the intensive surveillance. Therefore, they might have come to the decision of CRRM within a shorter period, in light of a current tumor diagnosis and therapy. In our interviews for the development of decision aids, survivors requested more information on BC and prognosis [30]. Communication of competing risks in advanced breast cancer cases is especially essential to avoid over-prevention.

Consistent with previous data, within the whole cohort, the preservation of the nipple did not lead to a higher satisfaction with outcome [34]. After nipple reconstruction, survivors with a more recent reconstruction had a significantly higher satisfaction with the nipple (*p* = 0.02; *n* = 19).

Within the survivors, we observed significantly higher scores in physical well-being (chest) after NSM/SCM compared to SSM with no further significant differences. The data situation is insufficient; other data demonstrated a significantly higher psychosocial well-being in after NSM compared to total mastectomy [34], or favorable trends for higher “satisfaction with breasts” and “satisfaction with outcome” in the SSM group compared with the NSM group [35]. In survivors, mean sexual well-being was significantly higher in NSM patients [36].

Most survivors were premenopausal with a mean age under 40. Chemotherapy may induce premature menopause with hot flushes, dry mucus membranes, and alopecia. Additional RRSO with a contraindication of hormone replacement therapy might have influenced QoL in survivors additionally [37]. Survivors after RRSO had a significant lower satisfaction with surgeon in the reconstruction module. We could not draw a causal reason for this result. Survivors stated a lower satisfaction with the surgeon. Depending on the tumor stage, adjuvant radiation is not necessary in the case of ablative surgery. In 36 cases, survivors decided on secondary mastectomy after BCT and radiation. There might be a regret about the decision for a two-stage procedure and/or an actual poorer outcome in IBR after radiation, as reported previously [38]. However, for survivors, the possible therapy-related side effects could have influenced satisfaction and QoL more than CRRM itself. Whereas endocrine therapy had no effect on any score of the mastectomy of reconstruction module, chemotherapy had a significant influence on satisfaction and QoL.

The significantly lower satisfaction of survivors after chemotherapy with the surgeon and medical staff underlines the increased consultation need among chemotherapy patients for care and counseling on preventive options, especially since actual satisfaction with the outcome did not differ significantly between survivors with or without chemotherapy.

Interestingly, Klapdor et al. demonstrated that patients with a high satisfaction with their breasts in the preoperative questionnaire had a higher risk of reduced postoperative scores, whereas patients who were less satisfied with the preoperative examination were more likely to have improved scores postoperatively [17]. High preoperative cancer distress predicts a negative body image [39]. Studies demonstrated that worse overall health status among survivors negatively influences satisfaction with breast reconstruction [40]. Therefore, it is conclusive that previvors had a significantly higher satisfaction in psychosocial well-being, sexual well-being, and physical well-being (chest) compared to survivors and is in accordance with previous data [41]. One might have expected otherwise while therapeutic mastectomies are accepted as a core component of cancer treatment and CRRM is in context with the cancer-affected breast surgery and therefore could be considered unavoidable.

To our best knowledge, this is the first study that compares postoperative BREAST-Q scores in previvors and survivors with *BRCA1/2* pV exclusively.

## 5. Conclusions

Independently of the diagnosis, probands with a higher score of psychological well-being had a higher satisfaction with information in the reconstruction module and a higher satisfaction with outcome.

The potential physical and psychological implications of this procedure have received much attention in the literature. A Cochrane review evaluated interventions to improve psychosocial well-being, such as mindfulness-based stress reduction [42]. Ho et al. showed that a higher satisfaction with information in survivors correlates with a higher satisfaction with the plastic surgeon and the surgical outcome [43].

Decision aids should therefore include medical and physical factors, psychological factors, and social context factors [31]. The patients experience of care, with office staff, medical team, and information, will enable sustainable decisions and, in the end, might lead to a higher satisfaction with outcome and QoL. It is to conclude that patients’ expectations for breast reconstruction play a key role in determining satisfaction with breast reconstruction and most likely with satisfaction with information.

Every *BRCA1/2* pV carrier should be informed about their BC risk and age-related incidences as well as satisfaction and QoL after RRM. Personalized risk prediction may support previvors and survivors in decision making, optimal time point for RRM, and long-term satisfaction with the chosen path. Younger age of onset of BC and additional indication for RRSO encouraged us to develop decision aids for pV carriers exclusively. Their clinical benefit is currently being evaluated.

## Figures and Tables

**Table 1:** Participants’ characteristics. Values are presented as mean ± SD. Carriers of pV Class 4 and 5 were included. Class 4: Probability of pathogenicity 0.95–0.99; Class 5: Probability of pathogenicity >0.99. Legend: ABR: autologous-based breast reconstruction, IBR: implant-based breast reconstruction, *n* = number, NSM/SCM nipple sparing/ subcutaneous mastectomy, SSM: skin-sparing mastectomy.

	Previvors *n* = 43	Survivors *n* = 90	All *n* = 133
*BRCA1* pV carriers	25	60	85
*BRCA2* pV carriers	18	30	48
Total	43	90	133
Age at BC (years) range (years)		39.6 ± 8 years 20–58	
Age at last RRM surgery (years) range (years)	36.4 ± 7.9 23–51	41.9 ± 8.2 21–60	40.1 ± 8.5 21–60
Follow-up time after last surgery (months)	43.3 ± 33.3	47.8 ± 50.2	46.3 ± 45.3
Method of mastectomy			
Bilateral NSM/SCM	26	8	32
Bilateral SSM	11	5	16
Combined/missing data on RRM	6	77	83
total	43	90	133
Method of reconstruction			
Bilateral ABR	7	14	21
Bilateral IBR	34	65	94
Combined methods/no reconstruction/missing data on RRM	2	11	13
Total	43	90	133
Age at RSSO (years) *n*	41.4 ± 6.2 *n* = 12	43.9 ± 6.41 *n* = 36	43.4 ± 6.5 *n* = 48

**Table 2:** Tumor phenotype and therapy of the 90 survivors with pV in *BRCA1/2.* Legend: c: clinical, ER: estrogen receptor, Her2: Human epidermal growth factor receptor 2, N: nodes, T: tumor, TNBC: triple-negative breast cancer.

Phenotype		Survivors *n*	Proportion of 90 Survivors (in%)
	Left side Right side	52 38	57.8 42.2
Tumor Stadium	cT1c	1	
	cT2	2	
	pT1	53	58.9
	pT2	20	22.2
	pT3	2	2.2
	T0	12	13.3
	Missing	3	3.3
	Total	90	100
Nodal Status	cN0	1	1.1
	cN1a	1	1.1
	pN0	63	70
	pN1 (1a, 1b)	20	22.2
	pN2	3	3.3
	pN3	1	1.1
	pNx	1	1.1
	Missing	1	1.1
	Total	90	100
Immunohistochemistry	ER pos	38	42.2
	ER neg	51	57.6
	Missing	1	1.1
	Total	90	100
	Her2 pos	9	10
	Her2 neg	78	86.7
	Missing	3	3.3
	Total	90	100
	TNBC	49	54.4
	TNBC in *BRCA1* pV	42	-
	TNBC in *BRCA2* pV	7	-
Systemic treatment	Neoadjuvant chemotherapy	35	38.9
	Adjuvant chemotherapy	46	51.1
	No chemotherapy	9	10
	Missing	1	1.1
	Total	90	100
	Endocrine therapy	32	35.6
	No endocrine therapy	58	64.6
	Total	90	100
Radiation	Adjuvant radiation	36	40
	No adjuvant radiation	54	60
	Total	90	100
	Adjuvant radiation after initial BCT	29/36	

**Table 3:** BREAST-Q Scores of the reconstruction module of all probands, previvors, and survivors. Values are presented as mean ± SD with consort diagram. *n* = number of participants with valid answers. Results in bold are statistically significant (*p* < 0.05).

	All *n*	Previvor *n*	Survivor *n*	*p*-Value
Satisfaction with breasts	66.9 ± 17.8 119	71.2 ± 16.6 38	64.8 ± 18.1 81	0.069
Satisfaction with outcome	78.3 ± 19.4 119	80.6 ± 20.1 39	77.2 ± 18.9 80	**0.0377**
Psychosocial well-being	73.7 ± 20.9 119	80.4 ± 19.5 39	70.5 ± 21.0 80	**0.015**
Sexual well-being	57.9 ± 21.7 112	67.1 ± 20.8 36	53.6 ± 20.9 76	**0.002**
Physical well-being chest	68.3 ± 15.9 121	76.1 ± 12.4 39	64.7 ± 16.2 82	**0.000**
Physical well-being abdomen	64.8 ± 25.9 29	55.0 ± 36.4 5	66.8 ± 23.5 24	0.361
Satisfaction with nipples	58.2 ± 28.0 25	58.7 ± 17.1 6	58.0 ± 31.1 19	0.964
Satisfaction with information	71.2 ± 17.4 120	76.9 ± 16.1 38	68.6 ± 17.4 82	**0.014**
Satisfaction with surgeon	86.9 ± 16.3 120	90.5 ± 14.2 39	82.2 ± 17.1 80	0.101
Satisfaction with medical staff	85.9 ± 19.9 118	87.7±18.3 38	85.0±20.7 80	0.505
Satisfaction with office staff	83.4 ± 20.0 117	84.6 ± 21.4 38	82.9 ± 20.6 79	0.670

## Data Availability

Data can be provided upon request.
